# Detection and Characterisation of SARS-CoV-2 in Eastern Province of Zambia: A Retrospective Genomic Surveillance Study

**DOI:** 10.3390/ijms25126338

**Published:** 2024-06-07

**Authors:** Doreen Mainza Shempela, Herman M. Chambaro, Jay Sikalima, Fatim Cham, Michael Njuguna, Linden Morrison, Steward Mudenda, Duncan Chanda, Maisa Kasanga, Victor Daka, Geoffrey Kwenda, Kunda Musonda, Sody Munsaka, Roma Chilengi, Karen Sichinga, Edgar Simulundu

**Affiliations:** 1 Churches Health Association of Zambia, Lusaka 10101, Zambia; jay.sikalima@chaz.org.zm (J.S.); karen.sichinga@chaz.org.zm (K.S.); 2 Virology Unit, Central Veterinary Research Institute, Ministry of Fisheries and Livestock, Lusaka 10101, Zambia; hermcham@gmail.com; 3 Global Fund to Fight AIDS, Tuberculosis and Malaria (GFATM), 1201 Geneva, Switzerland; fatim.jallow@theglobalfund.org (F.C.); michael.njuguna@theglobalfund.org (M.N.); linden.morrison@theglobalfund.org (L.M.); 4 Department of Pharmacy, School of Health Sciences, University of Zambia, Lusaka 10101, Zambia; steward.mudenda@unza.zm; 5 University Teaching Hospital, Ministry of Health, Lusaka 10101, Zambia; duncanchanda@gmail.com; 6 Department of Epidemiology and Biostatistics, School of Public Health, Zhengzhou University, Zhengzhou 450001, China; kasangaanita@gmail.com; 7 Public Health Department, Michael Chilufya Sata School of Medicine, Copperbelt University, Ndola 21692, Zambia; dakavictorm@gmail.com; 8 Department of Biomedical Sciences, School of Health Sciences, University of Zambia, Lusaka 10101, Zambia; kwenda.geoffrey@unza.zm (G.K.); s.munsaka@unza.zm (S.M.); 9 Zambia National Public Health Institute, Ministry of Health, Lusaka 10101, Zambia; kunda.musonda@znphi.com (K.M.); roma.chilengi@znphi.co.zm (R.C.); 10 Department of Disease Control, School of Veterinary Medicine, University of Zambia, Lusaka 10101, Zambia; 11 Macha Research Trust, Choma 20100, Zambia

**Keywords:** SARS-CoV-2, SAR-CoV-2 mutations, Omicron variant, Eastern Province, Zambia, genomic surveillance

## Abstract

Mutations have driven the evolution and development of new variants of the severe acute respiratory syndrome coronavirus 2 (SARS-CoV-2) with potential implications for increased transmissibility, disease severity and vaccine escape among others. Genome sequencing is a technique that allows scientists to read the genetic code of an organism and has become a powerful tool for studying emerging infectious diseases. Here, we conducted a cross-sectional study in selected districts of the Eastern Province of Zambia, from November 2021 to February 2022. We analyzed SARS-CoV-2 samples (*n* = 76) using high-throughput sequencing. A total of 4097 mutations were identified in 69 SARS-CoV-2 genomes with 47% (1925/4097) of the mutations occurring in the spike protein. We identified 83 unique amino acid mutations in the spike protein of the seven Omicron sublineages (BA.1, BA.1.1, BA.1.14, BA.1.18, BA.1.21, BA.2, BA.2.23 and XT). Of these, 43.4% (36/83) were present in the receptor binding domain, while 14.5% (12/83) were in the receptor binding motif. While we identified a potential recombinant XT strain, the highly transmissible BA.2 sublineage was more predominant (40.8%). We observed the substitution of other variants with the Omicron strain in the Eastern Province. This work shows the importance of pandemic preparedness and the need to monitor disease in the general population.

## 1. Introduction

Coronaviruses (family *Coronaviridae*) are the largest group of the order Nidovarales and can be present in humans and different animal species [1,2]. Emerging and re-emerging coronaviruses threaten global public health and the socio-economic well-being of populations [3,4]. The emergence of the severe acute respiratory syndrome coronavirus (SARS-CoV) in 2003 and the Middle Eastern respiratory syndrome CoV (MERS-CoV) in 2012 were of serious public health concern [5]. Recently, the SARS-CoV-2, a novel beta coronavirus that is genetically distinct from the SARS-CoV and MERS-CoV, has been responsible for the largest global health crisis to date [6,7].

Since the first case of coronavirus disease 2019 (COVID-19) in Wuhan, China in 2019, the SARS-CoV-2, the causative agent for COVID-19, has continued to evolve, acquiring mutations with the potential for evading host immune response [8,9,10,11]. In early 2020, the SARS-CoV-2 appeared to evolve relatively slowly for an RNA virus, and it was believed that the proposed vaccines would effectively control infections. However, by the end of December 2020, a highly mutated variant of concern (VOC), the Alpha variant, was first reported in the United Kingdom and spread globally [12]. The Alpha variant was responsible for driving the second wave of the pandemic. Among the notable mutations of the Alpha variant was the N501Y mutation in the S gene, which was associated with the increased binding affinity of the SARS-CoV-2 to the angiotensin-converting enzyme 2 (ACE2) receptor [13]. Similarly, a H69del/V70del mutation of the Alpha variant was associated with immune evasion and S gene target failure (SGTF) [14,15]. Consequently, the S gene target failure was used as a marker of the Alpha variant B.1.1.7 [15]. Similar to what was observed with the Alpha variant, the Beta and Gamma VOC were characterized by mutations in the S gene that resulted in increased transmission and potential for immune escape [14,16,17,18]. The Delta VOC, responsible for the third wave of the pandemic, became the dominant lineage globally and was characterized by the T478K S gene mutation, which resulted in increased virus binding affinity to ACE2 receptors [19].

In early November 2021, the Network for Genomic Surveillance in South Africa (NGS-SA) reported the emergence of a new and rapidly spreading variant, designated as the Omicron (B.1.1.529) VOC by the World Health Organization (WHO) [20]. The Omicron variant was remarkably genetically distinct, exhibiting over 40 amino acid residue changes in the spike protein [21]. The mutations were reported to increase transmissibility, high viral binding affinity and high potential for immune escape [22,23,24]. The increased viral fitness of the Omicron VOC was responsible for the fourth global wave of the SARS-CoV-2 pandemic [25,26]. The emergence of VOCs (Alpha, Beta, Gamma, Delta and Omicron) and variants of interest (VOIs; Epsilon, Eta, Iota, Kappa, Zeta and Mu) in the recent past highlights the continuous evolution of the SARS-CoV-2 [27,28]. Similar to what has been observed with the Omicron VOC, it is anticipated that the further evolution of the SARS-CoV-2 will result in variants with potential for antigenic shift, antigenic escape and increased transmissibility [29]. For instance, the B.1.351 (Beta) variant in Zambia was shown to coincide with a sharp increase in confirmed cases between December 2020 and January 2021 [30].

Genomic analysis has proved vital in understanding the continuous evolution of the SARS-CoV-2 [31,32]. However, the slow generation of genomic surveillance data in resource-limited countries like Zambia underlines the knowledge gaps that exist in SARS-CoV-2 surveillance. While recent genomic surveillance efforts have shed some light on the molecular epidemiology of SARS-CoV-2 in Zambia [30,33,34], there is still a paucity of information. This presents a missed opportunity for the early detection of emerging VOCs and VOIs. Moreover, the WHO emphasized the need to conduct the genomic surveillance of pathogens with epidemic and pandemic potential [35]. The Government of the Republic of Zambia has increased its capacity to conduct genomic surveillance for emerging and re-emerging pathogens with pandemic potential [12].

In this study, we performed genomic surveillance for SARS-CoV-2 in humans in selected districts in the Eastern Province of Zambia from November 2021 to February 2022.

## 2. Results

### 2.1. Demographic Summary

In total, 115 deidentified samples positive for SARS-CoV-2 on the rapid antigen test were submitted to the Churches Health Association of Zambia (CHAZ) laboratory for whole genome sequencing (WGS). The samples were collected from selected districts in the Eastern Province of Zambia. Of these, 58.3% (67/115) were obtained from female participants, while 40.2% were collected from male participants. Three samples (2.6%) had missing data. Most of the samples (33.0%) were collected from the 10–19 years age group, signifying a substantial representation of young individuals among SARS-CoV-2 cases, while only eight samples were collected from those who were above 50 years old. Twenty-eight samples (24.3%) had missing demographic data. The majority of the samples 37.4% (43/115) were obtained from Nyimba District while the remaining samples were obtained from Mambwe 18.3% (21/115), Chipangali 13.9% (16/115), Katete 12.2% (14/115), Lundazi 10.4% (12/115) and Chadiza 7.8% (9/115) Districts. The mean number of samples collected per district was 19.2% (Table 1).

### 2.2. SARS-CoV-2 Genome Detection and Assembly

Of the 115 samples analyzed by RT-qPCR, 35 (30.4%) were positive on three genes (ORF1ab, N, S), while the rest were positive on two genes (ORF1ab, N) (Table 2). For Whole Genome Sequencing (WGS) sample inclusion criteria, samples with Ct values ≤ 30 on either the ORF1ab, N or S gene targets were acceptable to undergo sequencing. Thus, all of the samples (*n* = 115; Ct < 30) were subjected to WGS on the Illumina NextSeq2000 platform. Raw reads generated by the Illumina NextSeq2000 platform were assembled into consensus sequences using the DRAGEN COVID Pipeline v1.1.0 available at https://emea.support.illumina.com/sequencing/sequencing_software/dragen-covid-pipeline/downloads.html (accessed on 1 February 2022). The non-gap ambiguity fraction rate ranged from 0 to 38.0% (average 10.3%). The mean sequence length was 29,664 (Std Dev: 233.7) while the average GC content was 37.9%. However, out of the 115 consensus sequences generated by the DRAGEN COVID Pipeline, 39 had stretches of more than 10% ‘NNNNNNNN’ and were thus removed from subsequent analysis. Thus, our final dataset of 76 sequences had an average sequence length of 29,752 (Std Dev: 27.5) and a non-gap ambiguity rate of 3%.

### 2.3. Genetic Diversity in SARS-CoV-2 Sequences

**Figure 1:**
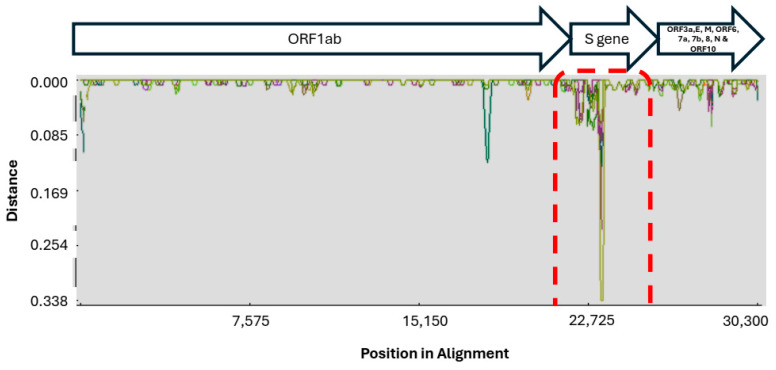
Genetic distance plot. Nucleotide diversity of SARS-CoV sequences from this study was calculated using the Wuhan HU-1 reference sequence (accession no. NC_045512).

Nucleotide diversity (π) was calculated across the genome in the 1 kb sliding window with a step size of 200 bp using the recombinant detection program (RDP4) and the Kimura two-parameter model on the nucleotide alignment generated by the MAFFT software [36,37]. There was a noticeable variation (0.00–0.337) in nucleotide diversity across the SARS-CoV-2 genome (Figure 1). Expectedly, high genetic diversity was observed in the S gene (area margined in with red dots), while the rest of the genes had relatively low genetic diversity.

**Figure 2:**
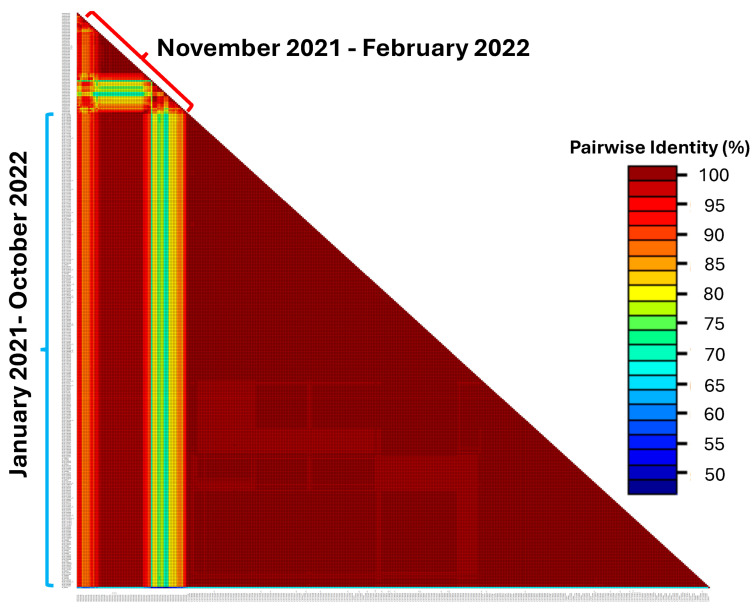
Pairwise genetic identity matrices of nucleotide sequences of the complete open reading frame of the SARS-CoV-2 spike protein. Viruses from this study (1 November 2021 to 28 February 2022). Reference sequences detected in Zambia between 1 January 2021 to 31 October 2021 are in black text denoted by blue right brackets. The colour indicates the homology level between sequences. Pairwise matrices were generated using the Sequence Demarcation Tool v.1.2 [38].

To further assess the diversity of SARS-CoV-2 strains in the present study, we used the Sequence Demarcation Tool (SDT) version 1.2 [38] to compare the complete nucleotide sequences of the S gene of SARS-CoV-2 strains from the present study to those detected in Zambia before 1 November 2021. In comparison to SARS-CoV-2 strains reported before 1 November 2021, viruses from this study were genetically diverse (Figure 2), suggesting the emergence of a highly divergent strain.

### 2.4. Synonymous and Non-Synonymous Mutations in SARS-CoV-2 Genomes

**Figure 3:**
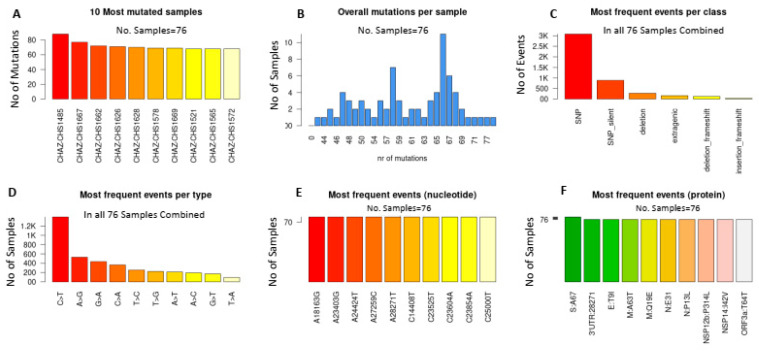
Mutation analysis of SARS-CoV-2 genomes in the present study. (**A**) Ten of the most mutated samples. (**B**) Number of overall mutations per sample. (**C**) Most frequently observed variant classifications. (**D**) Most frequently encountered substitution type. (**E**) Frequently observed nucleotide substitutions. (**F**) Most frequently observed amino acid mutations. Analysis was performed on 69 complete unique sequences using the Coronapp [39].

With respect to the Wuhan HU-1 reference sequence (accession NC_045512.2), we identified a total of 4097 mutations in the 69 unique SARS-CoV-2 genomes from the present study. Ten of the most mutated samples had at least 60 mutations (Figure 3A,B), while the most encountered events (>2500) were single nucleotide polymorphisms (SNPs) (Figure 3C). Similarly, the C > T transition was the most commonly encountered mutational event (>1200 events; Figure 3D). The most frequently encountered nucleotide substitutions were A18163G, A24424T, A28271T, C23525T and C23854A (Figure 3E), whereas the S: A67, E: T9I, M: Q19E, N: P13L and NSP14:I42V amino acid substitutions were observed in all the analyzed samples (Figure 3F).

A comparative analysis of amino acid sequences of the complete spike protein revealed remarkably high mutation frequencies. A total of 1925 mutations and 83 variant classes were identified in the spike protein. The spike protein accounted for the majority (47.0%; 1925/4097) of the observed mutational events (Table 2). High mutation counts (>100 count) were observed in the NSP3, NSP4, NSP5, NSP6, NSP12b, ORF3ab and M proteins. However, NSP2, NSP16, ORF7a, ORF8 and ORF10 had a low mutational count (<20 counts), suggesting that these genes are relatively stable (Table 3).

**Figure 4:**
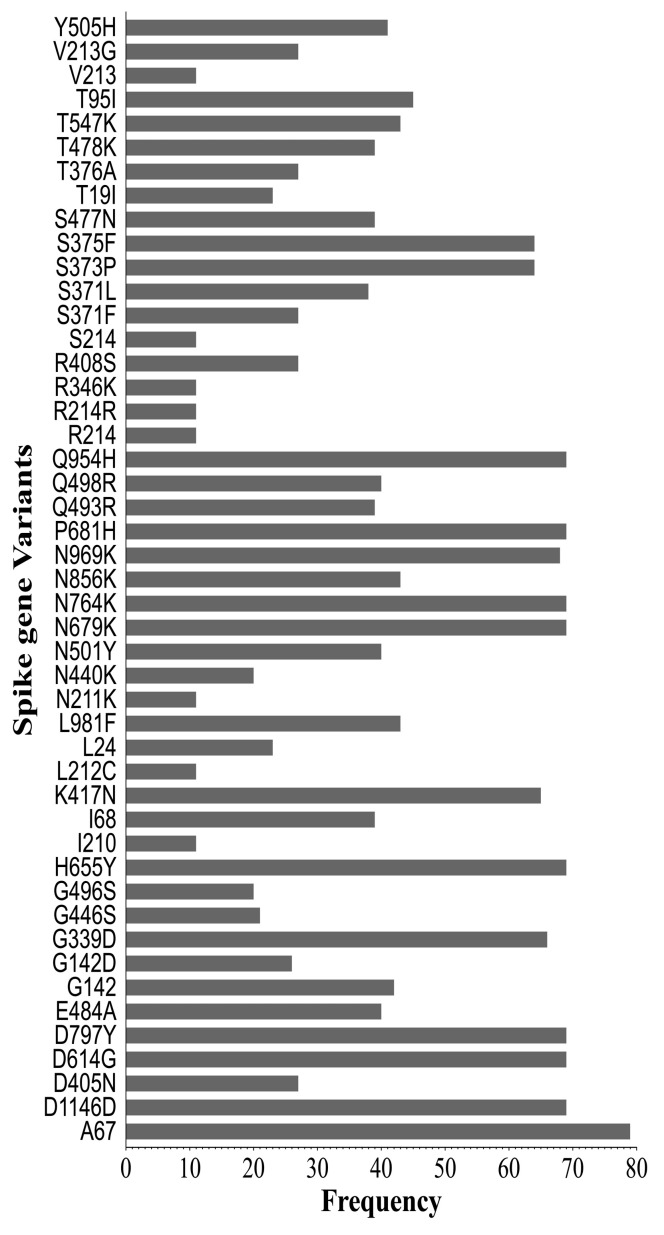
Frequency of observed variants in the spike protein of SARS-CoV-2 strains from the present study. The dotted blue line denotes the total number of analyzed samples (*n* = 69). Analysis was performed on the complete S protein of 69 unique sequences using the Coronapp [39].

Of the 83 variant classes, nine (i.e., Q954H, P681H, N764K, N679K, H655Y, D797Y, D614G, D1146D and A67) were present in all of the analyzed samples, while 41% (34/83) (A163A, A27S, A372, A397A, A475A, A845S, H69, I410V, I434M, K182K, K986T, L212L, L223L, L368I, N1098N, N211D, N234S, N370S, R190G, R237G, R328G, R403G, R454R, S399S, T333A, T376, T385A, T430A, T881T, V1264V, V367I, Y160C, Y170C and Y200C) were unique to only one sample (Figure 4).

In the receptor binding domain (RBD), the most predominant variants (>19 counts) were G339D, K417N, S375F, S373P, Y505H, E484A, Q498R, N501Y, S477N, T478K, Q493R, S371L, D405N, T376A, S371F, R408S, G446S, G496S and N440K (Table 4). The least encountered variants (<12 count) were R346K, T547K, A372, L368I, N370S, T430A, A397A, T376, I410V, T385A, I434M, R403G, S399S, V367I, A475A, R454R and T333A. Within the receptor binding motif, 12 variant classes, that is, Y505H, Q498R, E484A, N501Y, T478K, S477N, Q493R, G446S, G496S, N440K, R454R and A475A were identified. Y505H, Q498R, E484A, N501Y, T478K, S477N, Q493R, G446S and G496S were the most encountered mutations (>19 count), while N440K, R454R, and A475A were rare variants (<3 count).

### 2.5. Highly Divergent Strain Rapidly Replaced Other Variants after 1 November 2021

**Figure 5:**
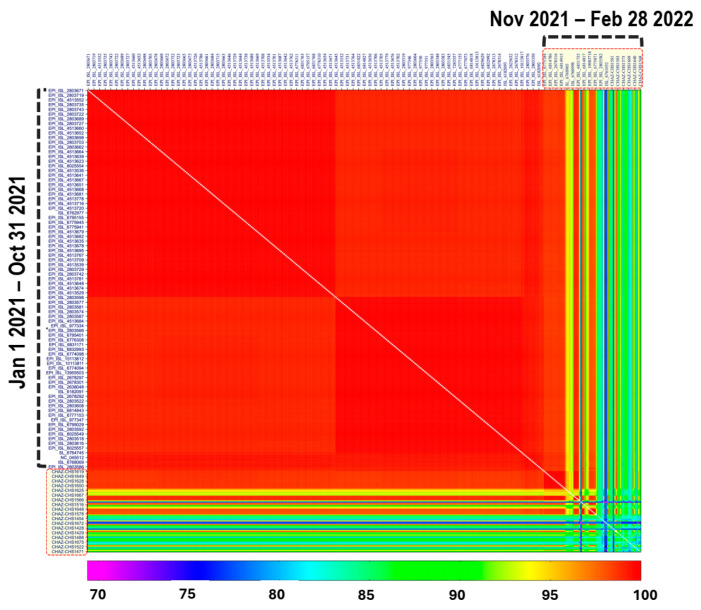
Pairwise nucleotide alignment of complete SARS-CoV-2 genomes from the present study and those downloaded from the GISAID database at https://www.epicov.org/epi3/ (accessed on 5 May 2024). Viruses from this study are in blue text and yellow highlight. Reference sequences are denoted in blue text. The horizontal bar denotes the percent similarity between sequences.

A pairwise matrix was generated in the Geneious R11 software using MAFFT (Figure 5) [37] available at https://mafft.cbrc.jp/alignment/server/ (accessed on 5 May 2024).

**Figure 6:**
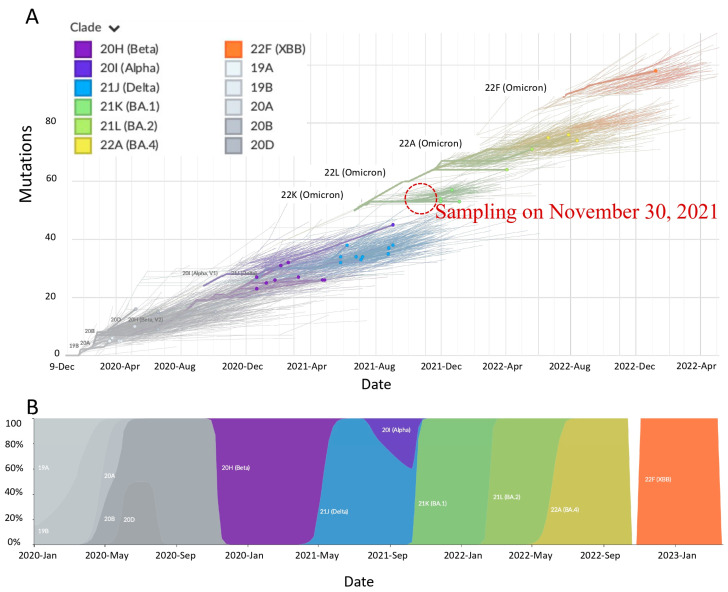
SARS-CoV-2 lineages identified in this study. (**A**) Frequency of SARS-CoV-2 Pango lineages identified in Eastern Province. (**B**) Distribution of SARS-CoV-2 Pango lineages in Eastern Province.

The pairwise nucleotide alignment of the complete SARS-CoV-2 genomes from this study and those downloaded from the GISAID database at https://www.epicov.org/epi3/ (accessed on 5 May 2023) showed that viruses detected after 1 November 2021 (this study) were genetically diverse as compared to those reported before 1 November 2021 (Figure 6). A time series analysis of the variants detected in Zambia so far showed the rapid replacement of other variants with the Omicron strain (Figure 6A,B). An inspection of nucleotide sequences deposited in the GISAID database shows that the first Omicron (BA.1, BA.1.17.2, and BA.1.19) samples in Zambia were collected 3 weeks (30 November 2021) after the first report of the Omicron variant in Botswana and South Africa [20]. Moreover, as of 4 February 2024, only Omicron and its subvariants have been reported from Zambia (GISAID; accessed 4 February 2024) so far. Notably, the early presence of the S gene target present (SGTP) BA.2 lineage in Eastern Province and other parts of the country resulted in some samples being misdiagnosed as Delta variant resurgence at the PCR stage. Taken together, our results suggest that the Omicron strain might have been present in Zambia earlier than previously thought. Likewise, the lack of detection of the other variants in samples collected after 1 November 2021 intimates that the Omicron variant was the dominant circulating strain.

### 2.6. Lineage, Clade Assignment and Recombination Analysis

**Figure 7:**
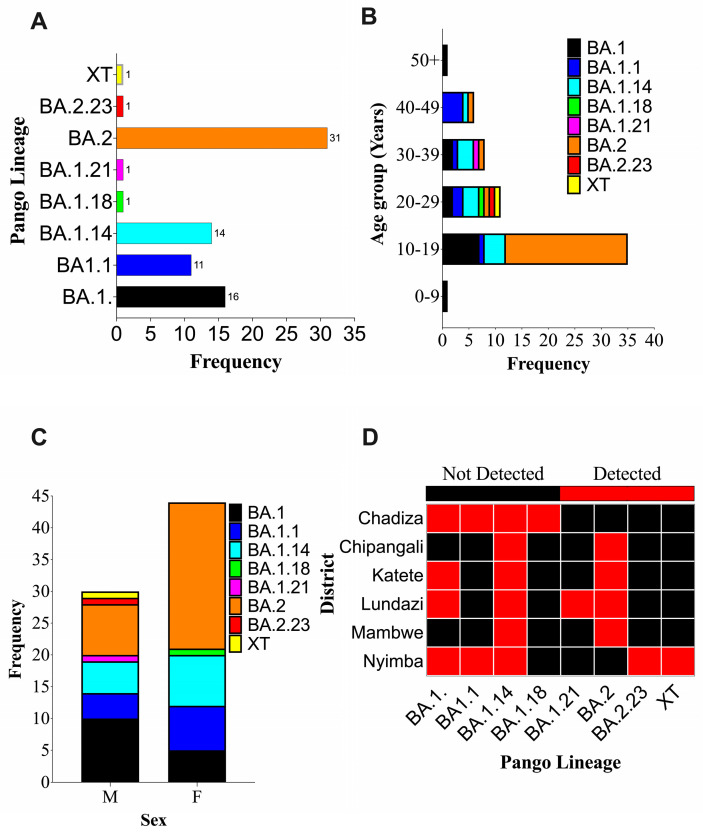
SARS-CoV-2 Lineages identified in this study. (**A**) Frequency of SARS-CoV-2 Pango lineages identified in Eastern Province. (**B**) Distribution of SARS-CoV-2 Pango lineages by age groups. (**C**) Distribution of Pango lineages by sex. (**D**) Detected Pango lineages by District.

The PANGOLIN [40] (https://pangolin.cog-uk.io/; accessed on 7 May 2023) and Nextclade [41] (https://clades.nextstrain.org; accessed on 7 May 2021) application softwares were used for lineage and clade classification. Samples (*n* = 76) from the present study were classified into eight Pango lineages, that is, BA.1, BA.1.1, BA.1.14, BA.1.18, BA.1.21, BA.2, BA.2.23 and XT. Interestingly, the XT strain (GSAID accession number EPI-1S1-10103848) was classified as a recombinant variant on the Nextclade analysis suit. Among the eight identified lineages, BA.2 was predominant, accounting for 40.8% (31/76) of all identified lineages (Figure 7A) while BA.1.21, BA.1.18, BA.2.23 and XT were the least (1.3%, 1/76) encountered lineages (Figure 7A). Intriguingly, the BA.2 lineage was more predominant in the 10–19-year-olds (Figure 7B) and also accounted for the majority of the identified lineages in female participants (Figure 7C). High lineage diversity was observed in 20–29-year-olds in whom all eight lineages (BA.1, BA.1.1, BA.1.14, BA.1.18, BA.1.21, BA.2, and BA.2.23) were identified (Figure 7B). With respect to lineage distribution, BA.1.14 was identified in all of the study sites, while the rare variants (BA.1.21, BA.1.18, BA.2.23 and XT) were detected only in Chadiza (BA.1.18), Lundazi (BA.1.21) and Nyimba (BA.2.23, XT) Districts. Furthermore, the eight lineages were classified into clades 21K (43/76) and 21L (32/76).

**Figure 8:**
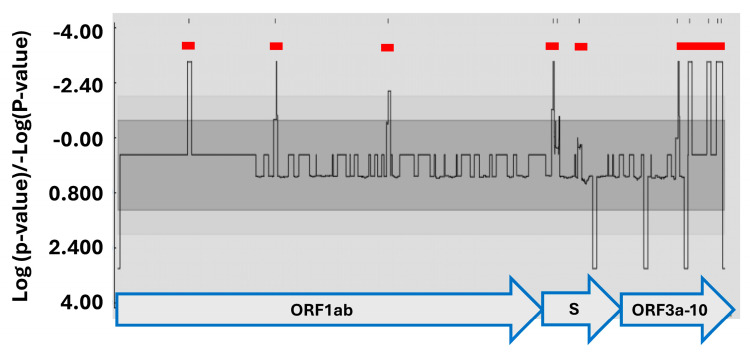
Detection of recombination hot/cold spots. Analysis was performed in RPD4 using a 200-base pair (bp) window at a 20-bp step and the Kimura two-parameter model on a nucleotide alignment generated by MAFFT. Recombination hotspots are denoted by red horizontal bars.

To account for the observed potential recombination event, (GSAID accession no. EPI–1S1–10103848), nucleotide sequences from this study together with those downloaded from the GISAID database were analyzed using the Recombination Detection Program version 4.101 (RDP4) [40]. Recombination breakpoints were detected in the ORF1ab, S, M and N (Figure 8).

### 2.7. Phylogenetic Analysis

On phylogeny, sequences from the present study formed a monophyletic group with other previously reported Omicron strains from Asia, Europe, America and Africa. This was suggestive of probable local and/or international transmission. Furthermore, Omicron strains shared a common ancestor with the Delta variant, exhibiting a descendant-like pattern. This finding supports earlier evidence of the emergence of the Omicron strain from the Delta variant. Topologically, Omicron strains from this study were classified into eight distinct lineages, that is, BA.1, BA.1.1, BA.1.14, BA.1.18, BA.1.21, BA.2, BA.2.23 and XT (Figure 8).

**Figure 9:**
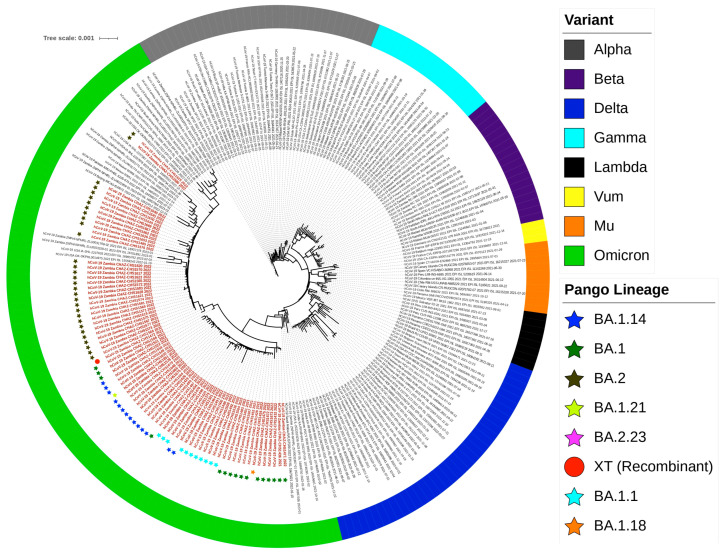
Maximum likelihood phylogenetic tree of SARS-CoV-2 genomes from Zambia and reference sequences retrieved from the GISAID database. The tree was implemented in IQ TREE [41] based on the best nucleotide substitution model (GTR + F+I + G4) in ModelFinder [42].

The phylogenetic tree was implemented in the IQ TREE [41] according to the best nucleotide substitution model (GTR + F+I + G4) in ModelFinder [42]. The reliability of the Phylogenetic tree was evaluated by 10,000 ultrafast bootstrap replicates [43]. Sequences generated in this study are denoted in red text, while reference sequences are in purple text. Coloured strips represent the SARS-CoV-2 variant. The coloured star denotes the assigned Pango Lineage. Bar, number of substitutions per site (Figure 9).

**Figure 10:**
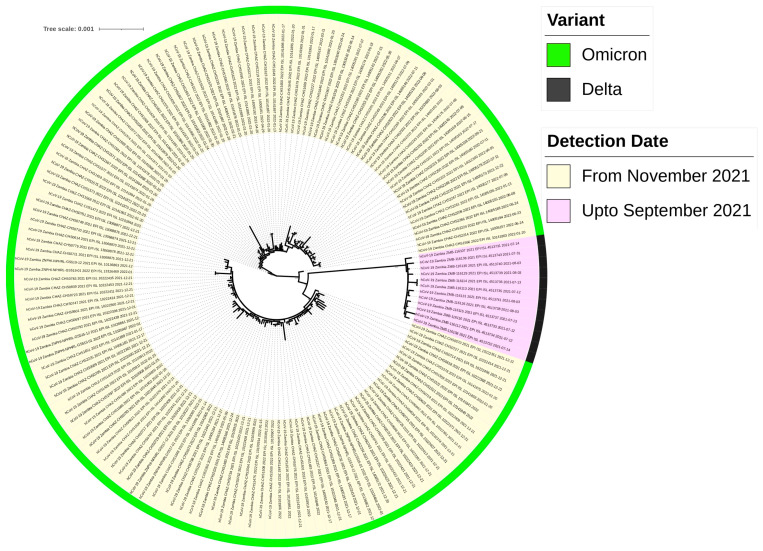
Maximum likelihood phylogenetic tree of SARS-CoV-2 genomes from the Eastern Province of Zambia collected between September 2021 and October 2022. The tree was implemented in IQ TREE [41] based on the best nucleotide substitution model (GTR + F+I + G4) in ModelFinder [42]. Phylogenetic tree reliability was evaluated by 10,000 ultrafast bootstrap replicates [43]. Coloured strips represent SARS-CoV-2 variants. Bar, number of substitutions per site.

Furthermore, the phylogenetic analysis of all samples collected in Eastern Province so far showed the rapid replacement of the Delta variant with the Omicron strain (Figure 10). This supports our earlier observation (Figure 5 and Figure 6) of the rapid replacement of the Delta variant after 1 November 2021.

## 3. Discussion

This study investigated mutations and their evolutionary relationships with other variants in the SARS-CoV-2 Omicron strain detected among residents of six selected districts in the Eastern Province of Zambia. This area was disproportionately affected by the surging numbers of COVID-19 infections at a time when numbers were receding in other provinces. We observed high genetic diversity in the spike protein. Similarly, high mutational counts were observed in the NSP3 to NSP6, NSP12b, ORF3ab and the M protein. Our findings are consistent with previous reports of high variations in the SARS-CoV-2 genome [44,45].

The present study revealed that there were more cases of the Omicron SARS-CoV-2 observed in females compared to males. Our findings are consistent with those of other studies that have reported a higher prevalence of the Omicron SARS-CoV-2 among females compared to males in Malawi, the United States of America and Zambia [34,46,47]. However, other studies have reported contrasting results, with more males being infected with the Omicron SARS-CoV-2 compared to females [48] with consistently more severe disease in males than in females [49,50]. These differences could be explained by the differences in transmission dynamics between the different settings, although our relatively limited sample size could also have influenced our findings.

The predominant age of patients was 10–19 years old, signifying a substantial level of SARS-CoV-2 infection among young individuals, while only eight samples were collected from those who were aged above 50 years old, indicating a lower prevalence of the infection in the older age group. Other studies have postulated that younger groups may drive COVID-19 infection due to frequent interactions such as during playing or school settings [51,52]. Despite the younger age groups being drivers of COVID-19 infection, they are less affected by the severe outcomes of the disease [51]. However, it cannot be ruled out that the small number of samples analyzed in this study may have had an impact on the observed gender distribution and age of COVID-19 patients, as was observed in a previous study [34].

This present study found that there was high genetic diversity in the S gene. High genetic diversity in the S gene has also been reported in other studies [53,54,55]. The areas of high diversity indicate highly mutated genes. Consequently, viruses, including the SARS-CoV-2, use mutations for evolution, survival, fitness and pathogenesis [53,54]. Our study found a total of 4097 mutations from 69 unique SARS-CoV-2 genomes, of which 1925 were in the spike protein. Additionally, 47% of mutational events were biased towards the spike protein, with high mutation counts observed in the NSP3, NSP4, NSP5, NSP6, NSP12b, ORF3ab and M proteins. Consequently, 83 variant classes were also obtained in the spike protein. These mutational events and variants indicate high diversity in the spike protein, similar to reports from other studies [56,57,58,59]. Unfortunately, the increased mutations in the spike protein of the SARS-CoV-2 Omicron variant may lead to the evolution of invasive and adaptive variants [21,60]. Additionally, a high frequency of single nucleotide polymorphisms (SNPs) was also found in our study, especially A18163G, A24424T, A28271T, C23525T and C23854A. Single nucleotide variants (SNVs) have also been reported elsewhere [61]. Similar findings were reported in a previous study in South and Southeast Asia, where high-frequency substitution sites were mainly localized in the S gene, including A24424T and C23525T [62]. The high frequency of these SNPs in a population may indicate genetic diversity, population stratification, disease prognosis or a lower prevalence of diseases and a higher risk of severe COVID-19 in certain individuals [63,64]. In South Africa, the Omicron variant had an average of 50 mutations, of which approximately 30 mutations were in the spike protein and about 15 in the RBD which affect virus transmission and immune escape [65]. Our findings and those reported in other studies imply that the continuous evolution of the S gene due to mutations threatens vaccine efficacy because the spike protein has been the main target of vaccine development [66,67].

The present study found 83 variant classes, of which 43.4% were located in the receptor binding domain (RBD) of the spike protein. These findings have also been reported in other countries [68,69,70,71]. This is a significant finding as the RBD is crucial for the interaction between the virus and the host cell receptor, ACE2 [13,72]. The presence of mutations in the RBD suggests potential impacts on viral high infectivity and immune recognition [21,73,74,75]. The stronger interaction between the RBD and ACE2 can cause Omicron to evade the antibodies that are produced by the COVID-19 vaccines [28,76,77]. This may render vaccines less effective against the SARS-CoV-2 Omicron variant [27,78]. The current study also identified specific variants that were more frequently encountered, including G339D, K417N, S375F, S373P, Y505H, E484A, Q498R, N501Y, S477N, T478K, Q493R, S371L, D405N, T376A, S371F, R408S, G446S, G496S and N440K. These variants may have functional implications, such as altered binding affinity to the ACE2 receptor or immune evasion, leading to the increased infectivity of the variants [71,73]. It is worth noting that some variants were rare, and these included R346K, T547K, A372, L368I, N370S, T430A, A397A, T376, I410V, T385A, I434M, R403G, S399S, V367I, A475A, R454R and T333A. Although these variants were less frequently encountered, they may still have important implications for viral fitness, immune evasion and pathogenicity.

The phylogenetic analysis of our study indicated that Omicron evolved independently from the Delta variant in the Eastern province, which has important implications for our understanding of the virus’s evolution and spread [79]. Notably, the Omicron variant emerged as the dominant strain from November 2021, while the Delta variant was scarcely detected. These findings resonate with findings from Puerto Rico, where the BA1 variant emerged replacing Delta as the dominant variant in December 2021, which was followed by increased transmission and a dynamic landscape of Omicron sublineage infections in the population [80]. Further studies have reported increased transmission and infection attributable to the emergence of the Omicron variant after the replacement of Delta in England, Finland and the United States of America [81,82,83]. Further, the spread of the Omicron variant in Eastern Province exhibited similarities to its spread in South Africa and Malawi [84,85]. This highlights the interconnectedness of regional transmission dynamics and emphasizes the importance of collaborative efforts in addressing the evolving landscape of SARS-CoV-2 variants [78].

We are aware that our study was conducted in one province of Zambia, thereby limiting the generalization of the findings to the rest of the country. However, our study suggests the co-circulation of some Omicron subvariants, which raises the risk of the generation of recombinants with potentially devastating mutations. We believe that this is the first genomic study on the SARS-CoV-2 in Eastern Province, Zambia, emphasizing the need for more comprehensive nationwide genomic studies to understand the evolution of the SARS-CoV-2 in Zambia. Additionally, the relative proximity of the Eastern province to Malawi poses a serious concern with cross-border transmission. Overall, the findings from this study contribute valuable insights into viral evolution patterns and highlight important considerations for genetic surveillance efforts aimed at monitoring changes in SARS-CoV-2 variants.

## 4. Materials and Methods

### 4.1. Study Area and Design

**Figure 11:**
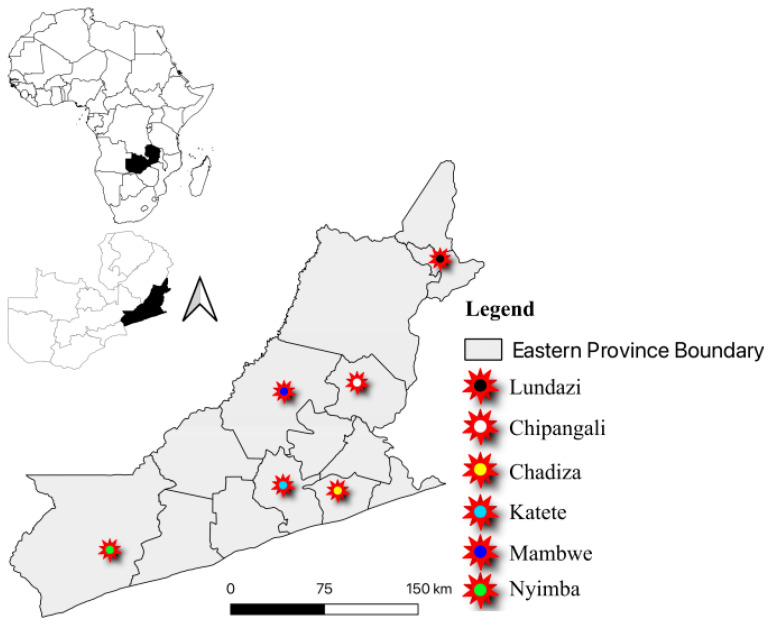
Map of Eastern Province showing the study sites of Chadiza, Nyimba, Katete, Chipangali, Mambwe and Lundazi Districts.

We conducted a cross-sectional study from November 2021 to February 2022 in the Chadiza, Nyimba, Katete, Chipangali, Mambwe and Lundazi Districts of the Eastern Province of Zambia (Figure 11). The Eastern Province of Zambia has a population of over 2.4 million people [86]. There is a high burden of HIV and Malaria in this region, and the proximity of the province to Malawi and Mozambique raises serious concerns about the cross-border spread of communicable diseases such as COVID-19.

### 4.2. Sample Collection and RNA Extraction

The criteria for next-generation sequencing (NGS) sample submission to the Churches Health Association of Zambia (CHAZ) laboratory were COVID-19 samples positive for the SARS-CoV-2 on the rapid antigen test kit. A total of 192 SARS-CoV-2 respiratory samples (i.e., nasal and throat swabs) positive for the SARS-CoV-2 antigen on the rapid test kit were included in this study. The samples were collected during routine surveillance and cluster outbreaks between November 2021 and February 2022. Sample collection was carried out using a flocked nasopharyngeal swab and stored on ice in virus transport media (COPAN Diagnostics, Inc., Murrieta, CA, USA) before being transported to the CHAZ Laboratory in Lusaka. Anonymized patient forms accompanying the samples were used to collect patient metadata including age, gender, place of residence, sampling date and clinical symptoms. These data were then entered into the DISA Laboratory Information and Management system (Laboratory System Technologies Ltd., Johannesburg, South Africa).

Viral RNA was extracted from nasopharyngeal swabs using the MagMAX viral isolation kit (Applied Biosystems, Foster City, CA, USA) on an automated Kingfisher Flex 96 Deep-well magnetic particle processor (ThermoFisher Scientific, Waltham, MA, USA) according to the manufacturer’s recommendation. Briefly, in a class II biosafety cabinet, a binding bead mix was prepared and aliquoted into 275 µL per sample well to which 200 µL of sample was added. Further, 20 µL of proteinase K/MS2 solution was added to the sample/binding bead mix and shaken at 1050 rpm for 2 min. Following the removal of the supernatant, samples were washed twice with wash buffer, and RNA was eluted in 50 µL of elution buffer for downstream processing.

### 4.3. SARS-CoV-2 Genome Detection by RT-qPCR

To confirm SARS-CoV-2 samples for whole genome sequencing and eliminate low copy number samples (sample cycle threshold (Ct) > 30), screening for the SARS-CoV-2 genome was conducted using the TaqPath COVID-19 CE-IVD RT-qPCR assay (ThermoFisher Scientific, Waltham, MA, USA) in a 25 µL reaction mix containing 15 µL of TaqPath qPCR master mix and 10 µL of extracted RNA.

### 4.4. cDNA Synthesis and Amplification of SARS-CoV-2

First-strand cDNA synthesis for samples was achieved through random hexamer priming using the First Strand cDNA master mix (Illumina) according to the manufacturer’s recommended protocol. Briefly, in a 96-well PCR plate, 8.5 µL of random hexamers were added to an equal volume of extracted RNA and denatured on an ABI 7500 real-time thermal cycler for 3 min at 65 °C. Ten µL of First Strand Mix and 1 µL of Reverse Transcriptase were then added to the denatured sample. cDNA synthesis was achieved with the following cycling conditions: 5 min at 25 °C, 10 min at 50 °C and 5 min at 80 °C. SARS-CoV-2 genome amplification was conducted using the ARCTIC network.

V4 primer pools ARCTIC V4 (https://github.com/artic-network/primer-schemes; accessed on 7 October 2021). The primer pool amplification employed two reactions per sample, i.e., COVIDseq Primer Pool 1 (CPP1) and COVIDseq Primer Pool 2 (CPP2). The reaction components for each reaction consisted of 12.5 µL of Illumina PCR Master Mix, 3.5 µL of either CPP1 or CPP2, 5 µL of first-strand cDNA synthesis and 3.9 µL of nuclease-free water. The thermoprofile was as follows: holding stage at 98 °C for 3 min, followed by 35 cycles of 98 °C for 15 s and 63 °C for 5 min. For each run, a single positive control (TaqPath COVID-19 Control; ThermoFisher, Carlsbad, CA, USA) and negative no template control (nuclease-free water) were included to serve as indicators of extraneous nucleic acid contamination. PCR amplicons for each sample were then combined by transferring 10 µL from each well of the CPP1 and CPP2 into a new well.

### 4.5. Library Preparation, Illumina Sequencing and Genome Assembly

Library preparation was performed using the Illumina COVIDSeq kit (Illumina Inc., San Diego, CA, USA) on the automated Hamilton robotic instrument (Hamilton, NV, USA). Pooled PCR products were processed for tagmentation and adapter ligation using the Illumina COVIDSeq Kit with IDT Illumina-PCR indexes. Pooling and library clean-up were performed as per the protocol provided by the manufacturer (Illumina Inc.). Pooled libraries were quantified on the Qubit 4.0 fluorometer (Invitrogen Inc., Waltham, MA, USA) using the Qubit dsDNA High Sensitivity kit. The pooled library was normalized to a 4 nM concentration. The library was further diluted to a final loading concentration of 1 nM using a resuspension buffer and sequenced (301 paired-end) on the Illumina NextSeq 2000 (Illumina, San Diego, CA, USA) platform.

To assemble SARS-CoV-2 whole genomes, the Illumina DRAGEN DNA pipeline was used to analyze sequence reads prepared using the ARCTIC gene panel assay (https://github.com/artic-network/primer-schemes; accessed on 7 October 2021). The DRAGEN pipeline uses a kmer reference database to match kmers from the sequencing read to kmers from the SARS-CoV-2 reference genome (Wuhan Hu-1, accession no. NC_045512). The kmer reference list is created by splitting the SARS-CoV-2 in 32bp kmers while any kmers that contain cross-reactivity are eliminated. Variant calling and consensus sequence generation were then performed for each sample using the DRAGEN COVID-19 pipeline. Consensus sequences were initially annotated using Glimmer in the Geneious software using the SARS-CoV-2 reference genome (NC_045512). Annotations were verified for reliability using genome annotation transfer utility (GATU) software [87].

Maximum likelihood phylogenetic analysis was implemented in IQ TREE [41] based on the best nucleotide substitution model in ModelFinder [42]. Phylogenetic tree reliability was evaluated by 10,000 ultrafast bootstrap replicates [43]. Tree editing and annotation were performed in the Interactive Tree of Life (iTOL) [88] software available at https://itol.embl.de (accessed on 7 May 2023).

### 4.6. Genetic Diversity and Mutations of SARS-CoV-2 Genomes

Analysis was performed in recombination detection program version 4 (RPD4) using a 200-base pair (bp) window at a 20-bp step and the Kimura two-parameter model on a nucleotide alignment generated by the MAFFT software. The analysis of synonymous and non-synonymous mutations was performed according to the complete Wuhan HU-1 reference strain (accession No. NC_045512.2). Mutation analysis in this study was performed on 69 complete unique sequences using the Coronapp, available at http://giorgilab.unibo.it/coronannotator/ (accessed on 7 May 2023). We conducted a comparative analysis of amino acid sequences of the complete spike protein to detect mutations and variants in the spike protein. The analysis was performed on the complete S protein of 69 unique sequences using the Coronapp, available at http://giorgilab.unibo.it/coronannotator/ (accessed on 7 May 2023). We further analyzed the Lineage, Clade Assignment and Recombination Analysis in the present study.

## Figures and Tables

**Table 1:** Demographics of participants who tested positive for SARS-CoV-2 in the Eastern Province of Zambia.

Description		N	%
			
Gender (*n* = 112)	Male	45	40.2
	Female	67	59.8
	Unknown	3	2.6
			
Age (*n* = 115)	0–9 years	3	3.4
	10–19 years	38	43.7
	20–29 years	18	20.7
	30–39 years	12	13.8
	40–49 years	8	9.2
	50+	8	9.2
	Unknown	28	24.3
			
District (*n* = 115)	Chadiza	9	7.8
	Chipangali	16	13.9
	Katete	14	12.2
	Lundazi	12	10.4
	Mambwe	21	18.3
	Nyimba	43	37.4

**Table 2:** Summary of TaqPath COVID-19 CE-IVD RT-qPCR assay.

Target			
S Gene	ORF1ab	N Gene	Number of Samples
−	+	+	80
+	+	+	35
		Total	115

**Table 3:** Mutation count of SARS-CoV-2 Strains with respect to the Wuhan HU-1 reference sequence (accession no. NC_045512).

Genomic Region	Mutation Count	Annotation
5′UTR	41	5′ Untranslated region
NSP1	27	RNA-dependent RNA polymerase
NSP2	8	
NSP3	378	
NSP4	156	
NSP5	134	
NSP6	122	
NSP9	39	
NSP10	43	
NSP12b	146	
NSP13	33	
NSP14	90	
NSP15	68	
NSP16	10	
S	1925	Spike
ORF3a	105	Open reading frame 3a protein
E	70	Envelope protein
M	198	Membrane protein
ORF6	96	Open reading frame 6 protein
ORF7a	5	Open reading frame 7a protein
ORF7b	59	Open reading frame 7b protein
ORF8	13	Open reading frame 8 protein
N	235	Nucleocapsid protein
ORF10	2	Open reading frame 10 protein
3′UTR	94	3′ Untranslated region
Total	4097	

**Table 4:** Mutational count in the receptor binding domain and receptor binding motif.

Receptor Binding Domain		
Variant Class	Count	%
G339D	66	1.6
K417N	65	1.6
S375F	64	1.6
S373P	64	1.6
Y505H	41	1.0
E484A	40	1.0
Q498R	40	1.0
N501Y	40	1.0
S477N	39	1.0
T478K	39	1.0
Q493R	39	1.0
S371L	38	0.9
D405N	27	0.7
T376A	27	0.7
S371F	27	0.7
R408S	27	0.7
G446S	21	0.5
G496S	20	0.5
N440K	20	0.5
R346K	11	0.3
T547K	6	0.1
A372	1	0.0
L368I	1	0.0
N370S	1	0.0
T430A	1	0.0
A397A	1	0.0
T376	1	0.0
I410V	1	0.0
T385A	1	0.0
I434M	1	0.0
R403G	1	0.0
S399S	1	0.0
V367I	1	0.0
A475A	1	0.0
R454R	1	0.0
T333A	1	0.0
Total	776	18.9
Receptor Binding Motif		
Y505H	41	1.0
Q498R	40	1.0
E484A	40	1.0
N501Y	40	1.0
T478K	39	1.0
S477N	39	1.0
Q493R	39	1.0
G446S	21	0.5
G496S	20	0.5
N440K	2	0.0
R454R	1	0.0
A475A	1	0.0
Total	323	7.9

## Data Availability

The data supporting the findings from this study are available from the corresponding author on request.
